# Quantitative Measurement of the Target-Mediated Internalization Kinetics of Biopharmaceuticals

**DOI:** 10.1007/s11095-014-1462-8

**Published:** 2014-09-11

**Authors:** Inna Vainshtein, Lorin K. Roskos, Jackie Cheng, Matthew A. Sleeman, Bing Wang, Meina Liang

**Affiliations:** 1 Clinical Pharmacology & DMPK, MedImmune LLC, Hayward, CA USA; 2 Respiratory, Inflammation and Autoimmunity, MedImmune Ltd, Cambridge, UK

**Keywords:** biopharmaceutical drug development, internalization kinetics, pharmacokinetic-pharmacodynamic modeling, image analysis

## Abstract

**Purpose:**

Measurement of internalization of biopharmaceuticals targeting cell surface proteins can greatly facilitate drug development. The objective of this study was to develop a reliable method for determination of internalization rate constant (k_int_) and to demonstrate its utility.

**Methods:**

This method utilized confocal imaging to record the internalization kinetics of fluorescence-tagged biopharmaceuticals in live-cells and a quantitative image-analysis algorithm for k_int_ determination. K_int_ was incorporated into a pharmacokinetic-pharmacodynamic (PK-PD) model for simulation of the drug PK profiles, target occupancy and the displacement of endogenous ligand.

**Results:**

The method was highly sensitive, allowing k_int_ determination in cells expressing as low as 5,000 receptors/cell, and was amenable to adherent and suspension cells. Its feasibility in a mixed cell population, such as whole blood, was also demonstrated. Accurate assessment of the k_int_ was largely attributed to continuous monitoring of internalization in live cells, rapid confocal image acquisition and quantitative image-analysis algorithm. Translational PK-PD simulations demonstrated that k_int_ is a major determinant of the drug PK profiles, target occupancy, and the displacement of endogenous ligand.

**Conclusions:**

The developed method is robust for broad cell types. Reliable k_int_ assessment can greatly expedite biopharmaceutical development by facilitating target evaluation, drug affinity goal setting, and clinical dose projection.

**Electronic supplementary material:**

The online version of this article (doi:10.1007/s11095-014-1462-8) contains supplementary material, which is available to authorized users.

## Introduction

Biopharmaceuticals targeting molecules on cell membranes are usually internalized into the cells. Target-mediated internalization and subsequently catabolism inside the cells represent an alternative route of elimination in addition to reticuloendothelial, hepatic, and renal clearance pathways [1–8]. Target-mediated clearance can result in rapid elimination from circulation and non-linear pharmacokinetics [9–14], which can affect drug exposure and ultimately efficacy. The efficacy of some drugs, such as immunotoxins and antibody-drug conjugates (ADCs), are dependent on the efficiency of target-mediated internalization to deliver the payload to cells [15–17]. Cell membrane targets with slow internalization rates are generally preferred for antibody therapeutics that are intended to elicit antibody-dependent cell-mediated cytotoxicity (ADCC) or complement-dependent cytotoxicity (CDC) [18–20]. A very high rate of receptor internalization on tumor cells can also create a barrier for drug penetration into solid tumors [20–22]. The internalization rate of the protein-receptor complex relative to the normal turnover rate of the receptor can result in post-treatment upregulation or downregulation of the receptor, which also can affect pharmacokinetics, potency, and efficacy.

Because internalization can affect the exposure and efficacy of biopharmaceuticals, assessment of internalization kinetics [23] is important during research and development. The internalization kinetics provide an important parameter for systems pharmacology and translational pharmacokinetic-pharmacodynamic (PK-PD) models [24, 25], which can be used to evaluate potential drug targets, establish protein engineering objectives for biopharmaceuticals and predict clinical dose requirements [26].

The primary challenge for quantitative measurement is direct monitoring of internalization events that allow a quantitative determination of the internalization kinetics of the drug-target complex. The majority of literature publications on receptor internalization present descriptive or qualitative approaches [27–32]. A few published quantitative internalization protocols such as acid dissociation and toxin-killing assays measure internalization indirectly. The acid dissociation method assesses antibody internalization by quantification of internalized molecules after acid removal of cell surface molecules [33, 34], whereas the toxin-killing method assesses internalization *via* cell-killing activity of internalized antibody-toxins [35]. Although both methods quantified the extent of internalization, they analyzed the outcomes or sequelae of internalization rather than internalization, *per se*.

A fluorescence-based imaging method provides a direct measurement of drug internalization kinetics. Fluorescence microscopy has been extensively used to visually demonstrate internalization of fluorescence-labeled receptors, ligands and antibodies [36–38]. Recent development of high-speed fluorescence microscopes equipped with live-cell chambers, laser-driven fluorochrome illumination and confocal capability provided highly sensitive systems for monitoring internalization in live cells. The advanced imaging technology combined with an algorithm for image analysis allowed development of a sensitive and robust method for quantification of the internalization rate constant. In this manuscript, we describe the development of this imaging method and the utility of the internalization kinetics in the discovery and development of biopharmaceuticals targeting cell membrane receptors.

## Materials and Methods

### Materials

TF-1, H1703, THP-1 cells were from ATCC. FD-hGMR cells transfected with human GM-CSF receptor were supplied by CSL Ltd (formerly Amrad, Melbourne Australia). Human whole blood from normal healthy donors was from Bioreclamation (Cat# HMWBNAHP, Westbury, NY). Mavrilimumab, MEDI-575 and isotype control IgG were from MedImmune. Anti-interferon alpha/beta receptor chain 2 monoclonal antibody (MMHAR2) was from PBL InterferonSource, Cat# 21385, Piscataway, NJ. Human recombinant GM-CSF was from R&D Systems (Cat# 215-g-010, Minneapolis, MN). RPMI (Cat# 11875–093), DMEM (Cat# 11965–092), Gibco® Cell Dissociation Buffer (Cat# 13151–014), 0.05% trypsin (Cat# 25300054), Hoechst 33342 (Cat# H1399), AlexaFluor® 488 (Cat# A-20181) and AlexaFluor® 647 (A-20186) Monoclonal Antibody Labeling Kits, CellTrace^TM^ proliferation kit CFSE (Cat# C34554) were from Life Technologies (Grand Island, NY). Fc receptor blocker was from Miltenyi Biotec Inc. (Cat # 120-000-442, Auburn, CA). Biogel P-6 gel purification resin was from Bio-Rad Laboratories, Inc, (Cat# 150–4130, Hercules, CA). CellCarrier 384-well microplates were from PerkinElmer Inc. (Cat# 6007550, Waltham, MA). Cedex HiRes automated cell counter was from Innovatis AG (Bielefeld, Germany). Confocal microplate fluorescent imager Opera was from PerkinElmer Evotec (Hamburg, Germany). BD Pharm Lyse buffer (Cat# 555899), BD FACS Canto II and BD Lyse Wash Assistant were from BD Biosciences, San Jose, CA, USA.

### Preparation of AlexaFluor Conjugates

Monoclonal antibodies, IgG isotype controls and recombinant proteins were conjugated with AlexaFluor-488 or AlexaFluor-647 dyes using the antibody labeling kits according to manufacturer instructions. In brief, 50–100 micrograms of an antibody or a protein in the sodium bicarbonate buffer, pH = 8.3, were incubated with reactive dye reagents under gentle agitation at room temperature for 1 h. Unincorporated dyes were removed by size exclusion chromatography using purification resins and columns provided in the kits. Fluorescent labeling of human recombinant GM-CSF was performed using the same fluorescent reactive reagents with 15 min conjugation time. Excesses of reactive materials were removed using a Biogel P-6, a purification resin for separation of GM-CSF with M.W. of approximate 14 kDa.

### Culture and Preparation of Cells for Staining

Adherent or suspension cells were cultured in T-75 flasks in the CO_2_ incubators using media recommended by ATCC or other suppliers. TF-1 cells were cultured in RPMI-1640 containing 10% fetal bovine serum (FBS) and 2 ng/mL of GM-CSF. FD-hGMR cells were cultured in DMEM containing 10% FBS and 5 ng/mL of GM-CSF. THP-1 cells were cultured in RPMI containing 10% FBS. Adherent H1703 cells were grown in RMPI containing 10% FBS. Cells were split according to manufacturer instructions and seeded for experiments.

Suspension cells were seeded using optimal cell densities a day before the experiment. For GM-CSF deprivation, TF-1 and FD-hGMR cells were cultured overnight in the respective cell media without addition of GM-CSF. Prior to staining, cells were washed twice with 1x phosphate buffered saline (PBS) using centrifugation at 1,200 rpm for 7–10 min, counted and resuspended at the concentration of 3×10^6^ cells/mL.

Adherent cells were stained using adherent or suspension protocols. For adherent protocols, cells were seeded into replicate wells in 384-well imaging plates and were grown in complete media one day before experiment. Seeding of 30,000-50,000 cells/well resulted in confluent monolayers after overnight culturing. Seeding densities were adjusted based on cell doubling times if cells were cultured over a longer period of time before experiments. On the experiment day, cells were washed twice with 1x PBS using gentle aspiration. Alternatively, adherent monolayers grown in T-75 flasks were dissociated into cell suspension using EDTA-dissociation buffer. Flasks with cells were washed twice with 1x PBS followed by 30 min incubation with 10 mL of dissociation buffer in the CO_2_ incubator. Detached cells were washed twice with 1x PBS using centrifugation at 1,200 rpm for 7–10 min. Cells were then resuspended into phenol-free RPMI plus 0.1% BSA at concentration of 3×10^6^ cells/mL.

Whole blood samples (70 µL) were stained with Hoechst 33,342 at 1 µg/mL in the CO_2_ incubator for 15 min. Samples were pre-chilled on ice and further incubated on ice with antibodies for 1 h. Erythrocytes lysis was then performed at room temperature with 1x BD Pharm Lyse buffer followed by 2–3 centrifugation washes with 1x PBS. Erythrocytes lysis and washes were carried out in the BD Lyse Wash Assistant. Resultant crude preparations of white blood cells were resuspended to the initial blood sample volumes using 1x PBS containing 0.2% BSA.

### Cell Staining and Analysis by Flow Cytometry

Cell suspension at 3×10^6^ cells/mL were pre-chilled on ice, blocked with the Fc receptor blocker for 5–10 min when needed and then incubated with the appropriate fluorescent conjugates for 1–2 h. In competition experiments, an unlabeled competitor molecule was pre-incubated with the cells for 30–60 min prior to the addition of the labeled counterpart. Following incubation on ice for 1–2 h, unbound fluorescent dyes were removed by 2–3 washes with ice-cold 1x PBS by centrifugation at 4°C. Cells were then resuspended in 1x PBS plus 0.2% BSA and immediately analyzed using flow cytometry on BD FACS Canto II. For multi-color analysis, spectral overlap was compensated using single dye-stained controls. A minimum of 2,500 events were acquired for cultured cell lines and 2,000 events in the monocyte gate for whole blood samples.

### Measurement of Mavrilimumab Internalization using Acid-dissociation Method

TF-1 cells at 3-6×10^5^ cells/mL were pre-blocked with FcR blocker on ice for 5–10 min and then incubated with 1 µg/mL of mavrilimumab-AlexaFluor-647 on ice for 1 h. Excess dye was removed by centrifugation and cells were either maintained on ice (4°C sample) or transferred to the CO_2_ incubator to initiate internalization (37°C sample). Internalization was carried out for two hours in the CO_2_ incubator. Each of the samples were split into two aliquots and treated with 1x PBS or acid (0.2 M acetic acid, 0.5 M NaCl, pH = 2.5) at 4°C for 10 min. Treated samples were washed twice with ice-cold 1x PBS by centrifugation, and mavrilimumab-AlexaFluor-647 fluorescence was measured by flow cytometry using BD FACS Canto II. Surface-localized mavrilimumab in each of the samples was determined from the decrease of AlexaFluor-647 signal by acid treatment. It was calculated using the equation 1-(FL_acid_/FL_PBS_), where FL_acid_ and FL_PBS_ were mavrilimumab-AlexaFluor-647 fluorescence signals of the sample in the presence and absence of acid, respectively. The 4°C sample was used as a “no internalization” control and thus was considered to have 100% of mavrilimumab-AlexaFluor-647 localized on cell surface. Therefore, internalized mavrilimumab-AlexaFluor-647 was calculated as the difference between the surface-localized mavrilimumab at 4°C and that at 37°C and expressed as percentage relative to the value of the 4°C sample.

### Cell Staining for Imaging

Cell suspension at 3×10^6^ cells/mL were incubated for 15–30 min with 1 µM CFSE prepared in 1x PBS at 37°C in the CO_2_ incubator. Unincorporated CFSE dye was removed by two 1x PBS washes using centrifugation at 1,200 rpm for 7–10 min at room temperature. Cells were then chilled on ice, blocked with the Fc receptor blocker if needed and stained with fluorescent antibodies or proteins as described for flow cytometry staining. After removal of unbound fluorescent reagents by centrifugation at 4°C, cells were resuspended in PBS containing 0.2% BSA or phenol red-free medium containing 1% FBS. Cells were transferred into multiple wells of 384-well imaging plate, and briefly centrifuged at 2,200 rpm for 2 min at 4°C prior to image acquisition.

Adherent cells were sequentially incubated at appropriate temperatures with CFSE, Fc receptor blocker if needed, and fluorescent antibodies directly in the wells of 384-well imaging plate. Excessive dyes were removed by gentle aspiration; 50–80 µL of imaging buffer (phenol red-free RPMI plus 1% FBS) were added to the wells of the 384-well plate prior to image acquisition.

For sodium azide treatment, CFSE-labeled cells were pre-treated with 0.1% sodium azide for 30 min prior to staining with fluorescent antibodies. Sodium azide was present during washes and incubations.

### Acquisition of Cell Images Using Opera

Stained cells in imaging plates (384-well format) were transferred into the “live-cell” chamber of the confocal fluorescence imager Opera under the controlled conditions of 37°C, 5% CO_2_ and 70% humidity to initiate and record internalization. A series of images were acquired at indicated times. Water-immersion objectives with 40x magnification, numerical aperture of 0.9 and 60x magnification, numerical aperture 1.2 were used for image acquisition.

Excitation lasers and emission filters for sample image acquisition were selected based on sample staining fluorochromes. Hoechst dye was excited by 405 nm laser and emission was collected in channel 1 (450 ± 50 nm), CFSE and FITC were excited by 488 nm laser and emission collected in channel 2 (540 ± 75 nm) and AlexaFluor-647 was excited by 635 nm laser and emission collected in channel 3 (690 ± 50 nm). If needed, dual laser excitations of 488/635 nm or 405/635 nm were used for multi-color stained samples. Prior to image acquisition in Opera, exposure parameters, such as voltages for laser excitations, exposure times, image binning factors, focusing height etc., were determined using an aliquot of the stained cells. Determined exposure parameters were applied to automatic image acquisition using Acapella 1.0 and Acapella 2.0 image acquisition software. Acquired image files were spooled over to the intermediate servers until retrieval for data processing. The raw images were exported as TIFF format and processed using ImageJ (NIH, Bethesda, MD) and Adobe Illustrator (Adobe, San Jose, CA).

### Algorithm for Image Analysis of Internalization

Antibody-associated fluorescence in membrane and cytoplasmic regions of cells residing in the field of image is quantified by an algorithm, which is a script for iterative processing of images acquired in reference (CFSE) and signal (antibody-AlexaFluor-647) channels of the imager. Images were processed by the algorithm using algorithm-defined parameters, which were initially set as default values and then optimized for each cell type and experiment.

The algorithm identifies cells as objects based on cytoplasm staining (CFSE channel), then it constructs “membrane” and “cytoplasm” regions around boundaries of cell objects and finally quantifies fluorescence signals within each area. The membrane region is generated around a cell object as a circular ring (for round suspension cells) or as an irregular shaped contour (for adherent cells) by extension of several pixels in and out of the boundary of the object. The remaining part of a cell object is denoted as cytoplasm. Since receptors tend to cluster into spots upon internalization, fluorescence intensity of spots was used to monitor antibody-associated AlexaFluor-647 signals. Fluorescent spots were identified based on intensity of fluorescence signal, its contrast to the background, the size of fluorescent spot and other characteristics using algorithm-based and user-adjustable parameters. The fluorescence intensities of the qualified spots in membrane and cytoplasm regions were quantified. Figure 2b shows selected steps of image analysis using the algorithm. CFSE staining of the image was identified as “Initial Mask (1)”. A threshold of fluorescence intensity was applied to exclude areas with fluorescence intensity signals below the threshold. The remaining CFSE-stained areas were segmented into cell objects, which were denoted as “Whole Cell (2)”. “Membrane Region (3)” and “Cytoplasm Region (4)” were constructed around the object boundaries using algorithm-defined parameters. The fluorescent spots were identified within each cell search region. Invalid spots (green – due to contrast, yellow – due to intensity and red – due to both) were rejected and valid spots (white) were accepted “Spot Detection (5)”. Accepted spots in membrane “Membrane Region Accepted Spot (6)” or cytoplasm “Cytoplasm Region Accepted Spot (7)” region are shown in arbitrary colors. Image analysis of adherent cells was conducted using the same algorithm as that for suspension cells with adjusting of cell-associated parameters to accommodate variable cell shapes, sizes, and fluorescence intensities.

Finally qualified membrane and cytoplasm spots were analyzed to derive the algorithm output values. The fluorescence intensities of membrane spots of all accepted cells were integrated and divided by sum of the pixel area of the membrane regions, which was used as the algorithm output value for the membrane signal. In a similar way, the cytoplasm signal output was derived by integrating fluorescence intensities of cytoplasm spots of the accepted cells and dividing by the sum pixel area of the cytoplasm regions. Several image fields per well were acquired to ensure sufficient number of cells for quantitative analysis. Typically, 40x lens magnification allowed capturing of 50–100 cells per image field for all cell types tested in this study.

Accumulation of antibody-associated fluorescence in the cytoplasm was used to quantify antibody internalization. To avoid under or over estimation of internalization rates relying strictly on signals in cytoplasm, cytoplasm signals were normalized by the total cell signal at each time point using the equation: Normalized Signal in cytoplasm = Signal (cytoplasm)/(Signal (cytoplasm) + Signal (membrane)). Normalization of the data ensured comparability of internalization results determined from samples with variable total cell fluorescence intensities due to staining variability. Internalization rate constants k_int_ were calculated from internalization time course by curve fitting of the data using the equation: ${S}_{cyt}(t)={S}_{0,\; cyt}+\left(1-{e}^{-{k}_{\mathrm{int}}\cdot t}\right)\cdot {S}_{\max,\;cyt}$, where *S*_*cyt*_ *(t)* is the cytoplasmic fluorescence signal at time t; *S*_*0,cyt*_ *(t)*and *S*_*max, cyt*_ were initial cytoplasmic fluorescence signal and maximal signal, respectively. The curve fitting of the data was conducted using SigmaPlot (Systat Software Inc., San Jose, CA). The half-life of internalization (T ½) was calculated as the ratio of ln2 and k_int_.

### Mechanistic Pharmacokinetic-pharmacodynamic Model

Structure of the mechanistic model is shown in Fig. 5. Disposition of an antibody and the endogenous ligand, interactions with the target receptor, and the internalization rate constant of antibody-receptor and ligand-receptor complexes are depicted by the differential equation system shown below:

1 $$ \frac{dAb}{dt}= Input-\frac{C{L}_{RES}}{V_c}\cdot Ab-\frac{k_{on}}{V_c}\cdot Ab\cdot R+{k}_{off}\cdot Ab R-\kern0.5em \frac{Q}{V_c}\cdot Ab\kern0.5em +\kern0.5em \frac{Q}{V_p}\kern0.5em \cdot \kern0.5em A{b}_p $$

2 $$ \frac{dA{b}_p}{dt}=\kern0.5em \frac{Q}{V_c}\cdot Ab\kern0.5em -\kern0.5em \frac{Q}{V_p}\kern0.5em \cdot \kern0.5em A{b}_p $$

3 $$ \frac{dL}{dt}={S}_L-\frac{C{L}_L}{V_C}\cdot L-\frac{k_{onL}}{V_C}\cdot L\cdot R+{k}_{offL}\cdot LR $$

4 $$ \frac{dR}{dt}={S}_0-{k}_{\operatorname{int},R}\cdot R-\frac{k_{on}}{V_c}\cdot Ab\cdot R+{k}_{off}\cdot Ab R-\frac{k_{on L}}{V_L}\cdot L\cdot R+{k}_{off L}\cdot LR $$

5 $$ \frac{dAbR}{dt}=\frac{k_{on}}{V_c}\cdot Ab\cdot R-{k}_{off}\cdot Ab R-{k_{\mathrm{int}}}_{, AbR}\cdot Ab R $$

6 $$ \frac{dLR}{dt}=\frac{k_{onL}}{V_C}\cdot L\cdot R-{k}_{off}\cdot LR-{k}_{\operatorname{int}, LR}\cdot LR $$

In Equation 1, *Input* represents the intravenous administration of the antibody into the central compartment. V_c_ and V_p_ are the central and peripheral distribution volumes, respectively. Q is the intercompartmental flow. CL_RES_ and CL_L_ are the systemic clearance of the antibody by the reticuloendothelial systems and the endogenous ligand, respectively. The association constants, k_on_ (for antibody) and k_onL_ (for ligand), were scaled by V_c_ as the unit of k_on_ and k_onL_ is related to the concentration instead of amount. S_L_ and S_0_ are the zero-order endogenous production rate of the ligand and the target receptor, respectively. The internalization rate constants k_int,R_, k_int,AbR_ and k_int,LR_ are for the unbound receptor R, the antibody-receptor complex AbR and the ligand-receptor complex LR, respectively.

Simulations were performed using software package NONMEM (Version 7.2, ICON Development Solutions, Ellicott City, MD). For illustration purpose neither inter individual variability nor assay residual error were incorporated in the model. The antibody disposition parameters CL_RES_ (0.186 L/day), V_c_ (3.06 L), V_p_ (1.77 L) and Q (0.294 L/day) were assumed the same as a typical IgG not subject to receptor-mediated clearance [39]. The endogenous ligand was assumed to have a baseline level of 0.01 nM, a serum half-life of 2 h and an affinity of 0.1 nM to the target receptor, typical for a soluble cytokine [40–43]. Upon single IV administration of the antibody, simulations were then performed for both slow- and fast-receptor-internalization scenarios with various assumed receptor expression levels (R_0_ = 0.1 or 0.6 nM) or antibody binding affinity (K_d_ = 0.1, 0.3 or 1 nM). The receptor expression level was based on analysis of clinical PK data of several mAbs against membrane-associated receptors. The binding affinity reflected the range of the *in vitro* experimental data generated at MedImmune for a number of mAbs. From confocal imaging studies, most antibody-bound receptors had an internalization T ½ of approximately 30 min (*e.g.*, GM-CSF receptor alpha and PDGF receptor alpha). On the other hand, the internalization of some receptors, such as IFNAR2, was much slower (~2 h). The translational simulations evaluated both internalization scenarios, with a typical internalization T ½ of 30 min or 2 h.

## RESULTS

### Development of an Image-based Method for Quantification of Internalization Kinetics

#### Measurement of Internalization

To measure internalization, a therapeutic antibody was labeled with a fluorescent tag, pre-bound to target cells and its translocation from cell surface to cytoplasm was monitored by fluorescence imaging. For the purpose of defining the cytoplasmic compartment, cells were stained with a second fluorescent dye. To ensure high image quality, fluorochromes for antibody and cytoplasm labeling were selected from the dyes with minimal spectral overlap. Therefore, blue or green dyes in combination with far-red dyes were considered for dual-color staining. We first evaluated optimal fluorochromes for antibody labeling. Commonly used AlexaFluor-488 (green) or AlexaFluor-647 (red) dyes were conjugated to mavrilimumab, a human IgG_4_ antibody directed against human GM-CSF receptor alpha. Binding specificity of mavrilimumab-AlexaFluor conjugates was examined in human monocytes, which express human GM-CSF receptor alpha. Supplementary Fig. 1 shows mavrilimumab-AlexaFluor-647 bound to CD14^+^ monocytes. The binding was completely blocked by pre-incubation with an excess amount of unlabeled mavrilimumab, demonstrating binding specificity. A similar result was observed for mavrilimumab-AlexaFluor-488 (data not shown). Signal intensities of mavrilimumab-AlexaFluor-488 and mavrilimumab-AlexaFluor-647 conjugates were compared in TF-1 cells expressing endogenous human GM-CSF receptor alpha and the engineered FD-hGMR cell line over-expressing the recombinant human receptor (Supplementary Table I). Both cells demonstrated 5–10 fold higher signals for mavrilimumab-AlexaFluor-647 than for mavrilimumab-AlexaFluor-488. In addition, AlexaFluor-647 dye is known to be less susceptible to photobleaching and the detection of its emission is minimally affected by the autofluorescence of the cells. Therefore, AlexaFluor-647 was selected for antibody labeling and cytoplasm was stained using a green channel emitting dye, carboxyfluorescein succinimidyl ester (CFSE).

**Fig. 1:**
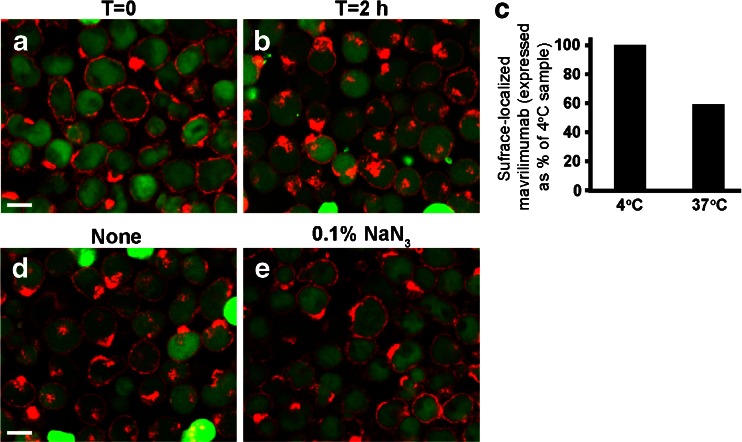
Assessment of mavrilimumab-AlexaFluor-647 internalization in TF-1 cells. (**a,b**) Overlays of CFSE (*green*) and AlexaFluor-647 (*red*) channels for cells stained with CFSE and 1 µg/mL mavrilimumab-AlexaFluor-647 prior to (**a**) and after incubation at 37°C for 2 h (**b**) are shown.(**c**) Levels of surface-localized mavrilimumab-AlexaFluor-647 in the control (4*°*C) and the internalization (*37*°C*, 2 h*) samples determined by an acid dissociation protocol (see Materials and Methods). Surface-localized mavrilimumab-Alexa-647 is shown relative to the value of the 4°C sample, which represented 100% of surface binding. (**d,e**) Inhibition of mavrilimumab-AlexaFluor-647 translocation to cytoplasm by sodium azide. Shown are overlays of CFSE (*green*) and mavrilimumab-AlexaFluor-647 (red) signals of cells treated without (**d**) or with 0.1% NaN_3_ (**e**) followed by 2 h incubation at 37°C. Scale bars, 10 µm.

Receptor-mediated internalization of mavrilimumab was assessed in TF-1 cells. Fig.1a, b shows cells labeled with CFSE and mavrilimumab-AlexaFluor-647 prior to and two hours post internalization. Image overlays of CFSE (green) and AlexaFluor-647 (red) demonstrated that AlexaFluor-647 signals were localized primarily on the cell surface before initiation of internalization (T = 0) and were associated with the cytoplasm area (green) after 2 h (T = 2 h) at 37°C. Translocation of mavrilimumab-AlexaFluor-647 fluorescence signal from cell surface to cytoplasm indicated receptor-mediated internalization.

To confirm that observed mavrilimumab-AlexaFluor-647 fluorescence translocation was due to internalization, the imaging result was compared with that using an acid-dissociation procedure. Because acid treatment of cells only removes antibody from the cell surface and does not affect internalized antibody in the cytoplasm, detection of fluorescence signal remaining after acid treatment serves as an indirect measurement of antibody internalization. TF-1 cells, pre-bound with mavrilimumab-AlexaFluor-647, were either maintained at 4°C or incubated at 37°C for 2 h. Each of the samples was treated with and without acid and analyzed by flow cytometry. The decrease in the fluorescence intensity following acid treatment was proportional to the amount of mavrilimumab on the cell surface. Figure 1c shows surface-localized mavrilimumab determined for the 4°C and 37°C samples. At 4°C, no or minimal internalization was expected and thus the value represented 100% of surface-localized mavrilimumab. The amount of surface-localized mavrilimumab after 2 h incubation at 37°C was approximately 60% as compared to that at 4°C, demonstrating antibody internalization. The percentage of internalized antibody calculated from the decrease of surface-localized mavrilimumab after 2 h at 37°C relative to the 4°C sample was approximately 40% (see Materials and Methods).

To further validate the image-based method, TF-1 cells were treated with sodium azide (NaN_3_), an inhibitor of cell metabolism and energy-dependent internalization. Treatment with NaN_3_ inhibited intracellular translocation of mavrilimumab-AlexaFluor-647 fluorescence signals, consistent with blockade of antibody internalization (Fig. 1d, e).

#### Measurement of Antibody Internalization Rate Constant (k_int_)

**Fig. 2:**
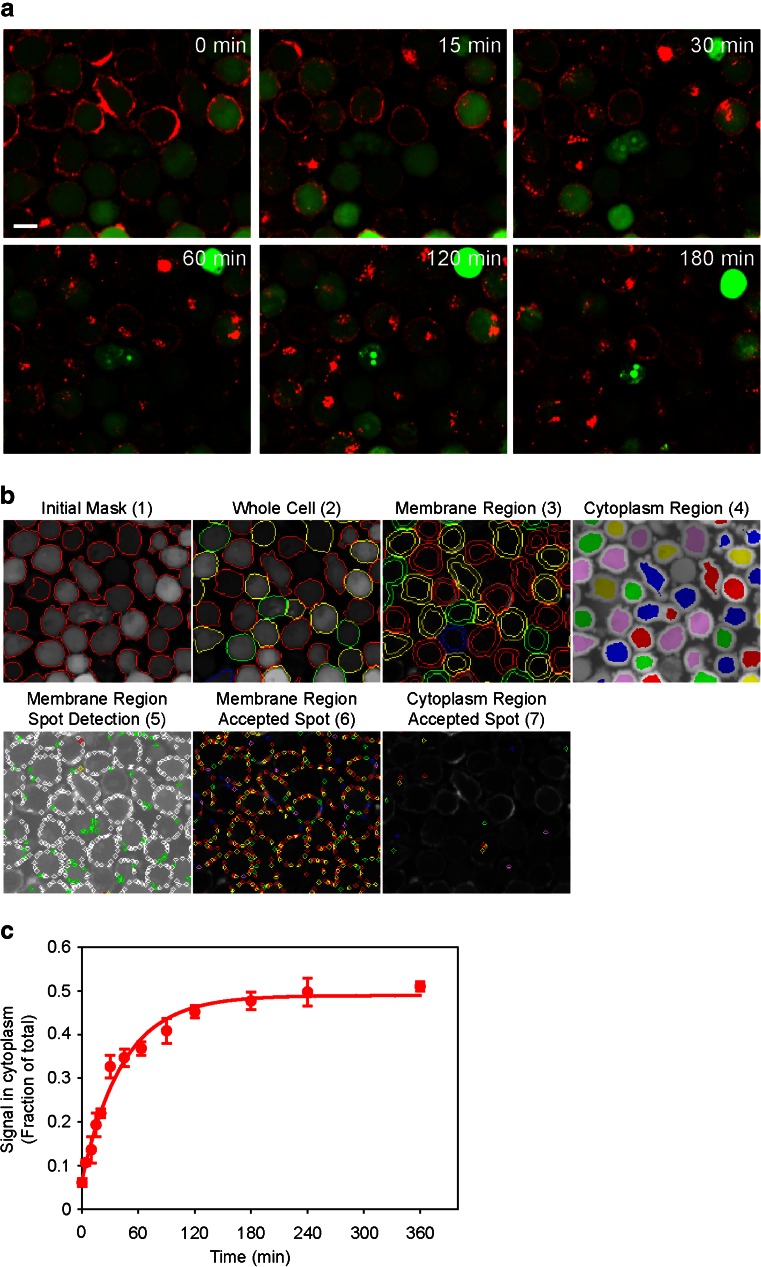
Measurement of mavrilimumab-AlexaFluor-647 internalization rate constant in TF-1 cells. (**a**) Time course of antibody internalization presented as an image sequence. Overlays of CFSE (*green*) and mavrilimumab-AlexaFluor-647 (*red*) image channels at indicated time points are shown. Scale bar, 10 µm. (**b**) Algorithm for image analysis demonstrated using T = 0 image. Representative steps of iterative analysis are depicted. Initial mask (1) – the entire area of CFSE staining. Whole Cell (2) – the area of CFSE signals above the threshold segmented into separate cell objects. Membrane Region (3) – the area around the boundaries of cell objects using algorithm-defined parameters. Cytoplasm Region (4) – the area between the internal membrane boundary and the center of the cell object. Membrane Region Spot Detection (5) - mavrilimumab-AlexaFluor-647 signals detected as fluorescent spots in the membrane region and classified based on size, signal intensity, contrast and other parameters. Accepted spots are shown in white, rejected based on intensity - in yellow, rejected based on contrast - in green, and rejected based on both intensity and contrast - in red. Membrane Region Accepted Spots (6) and Cytoplasm Region Accepted Spots (7) – qualified spots in the membrane and the cytoplasm areas (shown in arbitrary colors). Outputs were spot fluorescence intensities in membrane and cytoplasmic regions. (**c**) The time course of mavrilimumab-Alexa-647 accumulation in cytoplasm. Algorithm-derived mavrilimumab-Alexa-647 signals at each time point are shown as normalized to total cell fluorescence. One of the two independent experiments is presented. Data are plotted as mean ± standard deviation of triplicate wells. Internalization rate constant (k_int_) was determined from curve fitting of the fluorescence data: ${S}_{cyt}(t)={S}_{0,\; cyt}+\left(1-{e}^{-{k}_{\mathrm{int}}\cdot t}\right)\cdot {S}_{\max,\;cyt}$, where *S*_*0,cyt*_ *(t)* and *S*_*max, cyt*_ are initial cytoplasmic fluorescence signal and maximal signal, respectively. T ½ calculated from k_int_ was 34 ± 5 min.

To measure k_int_, the kinetics of cell-bound antibody translocation from cell surface to cytoplasm was continuously monitored by fluorescence imaging. A series of cell images taken over time course of mavrilimumab-AlexaFluor-647 internalization in TF-1 cells is shown in Fig. 2a. Image overlays of CFSE (green) and AlexaFluor-647 (red) demonstrated predominate mavrilimumab-AlexaFluor-647 localization on the cell surface at time zero and time-dependent translocation of the signal into cytoplasm over three hours at 37°C.

Acquired kinetic images were analyzed with a custom-developed algorithm. Iterative steps of image analysis for CFSE and antibody (AlexaFluor-647) channels were described in detail in the Methods section and briefly summarized in Fig. 2b. The algorithm first identified cells as objects based on boundaries of cytoplasm staining (CFSE channel), then defined “membrane” and “cytoplasm” regions around contour of cell objects and finally quantified fluorescence intensity within each region. Since receptors tend to cluster upon internalization, fluorescence intensity of the spots was quantified.

Algorithm-derived mavrilimumab-AlexaFluor-647 signals in cytoplasm over the time course of internalization are shown in Fig. 2c. To minimize variability of fluorescence staining between samples and experiments, the cytoplasm fluorescence signals were normalized to total cell fluorescence. The internalization rate constant was determined from the curve fitting of the data shown in the time course. The T ½ of mavrilimumab-AlexaFluor-647 internalization derived from k_int_ was 34 ± 5 min. The extent of internalization shown in Fig. 2c was consistent with that determined by the acid dissociation method in Fig. 1c (approximately 40% at 2 h).

### Versatility and Robustness of the Method

#### The Method is Suitable for Suspension and Adherent Cells

**Fig. 3:**
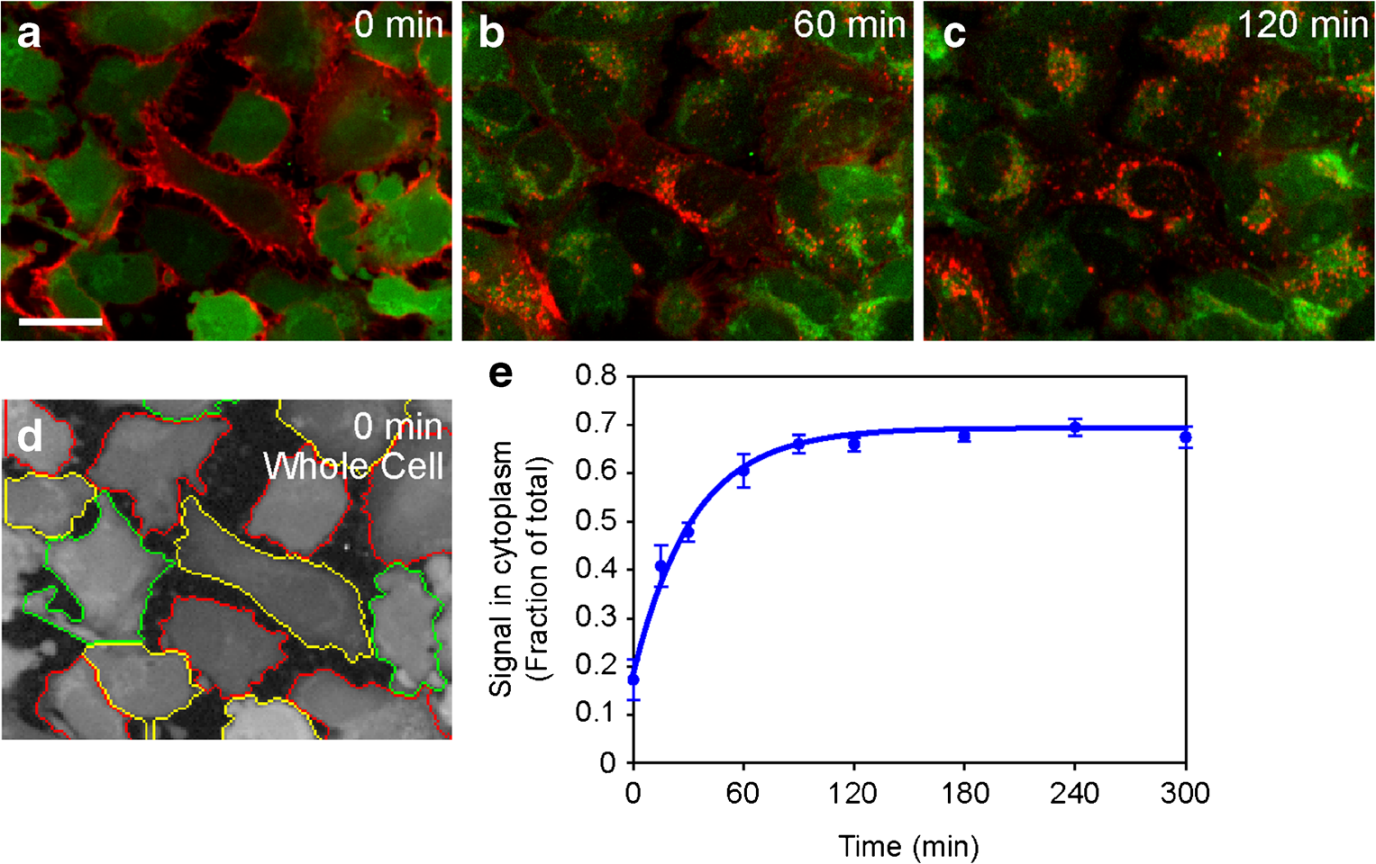
Antibody internalization in adherent cells. (**a-c**) Internalization of MEDI-575-AlexaFluor-647 (1 µg/mL) in H1703 cells stained with CFSE is shown at T = 0 (**a**), T = 60 min (**b**) and T = 120 min (**c**). Displayed are image overlays of CFSE (*green*) and MEDI-575-AlexaFluor-647 (*red*). Scale bar, 25 µm. (**d**) Object identification in adherent cells by the algorithm. The algorithm utilized similar iteration steps as that used for suspension cells (shown in Fig. 2b). The cell sizes and shapes were adjusted using user-defined parameters. The step of the algorithm, Whole Cell, which depicts identification of irregular-shaped cell objects in adherent cells (T = 0 image), is shown. (**e**) Time course of MEDI-575-AlexaFluor-647 internalization in H1703 cells quantified by the adherent cell algorithm. One of the six independent experiments is presented. Data are plotted as mean ± standard deviation of triplicate wells. Internalization rate constant (k_int_) was calculated using equation shown in figure legend to Fig. 2c. T ½ was 23 ± 2 min.

The method and algorithm was originally developed for cells in suspension. The suitability of the method was examined for adherent cells. The adherent cells are usually more heterogeneous in sizes and shapes, leading to difficulty in object segmentation for image analysis. Internalization of MEDI-575, a monoclonal antagonistic antibody to human PDGF receptor alpha, was examined in adherent cell line H1703 (a non-small cell lung cancer cell). Figure 3a-c shows cytoplasmic translocation of MEDI-575-AlexaFluor-647 over time at 37°C. Algorithm parameters were adjusted to adapt for irregular shapes and cell size heterogeneity of adherent cells (Fig. 3d). Kinetic images taken over the time course of MEDI-575-AlexaFluor-647 internalization were analyzed using the algorithm. The internalization rate constant was determined from curve fitting of MEDI-575-AlexaFluor-647 accumulation in cytoplasm (Fig. 3e). T ½ derived from k_int_ was 23 ± 2 min. Measurement of MEDI-575 internalization in H1703 cells using an alternative suspension protocol, where adherent cells were detached from the plates prior to staining and initiation of internalization, demonstrated comparable internalization T ½ (30 ± 8 min). The results indicated that the method and the algorithm were robust and amenable to both adherent cells and suspension cells.

#### The Method is Suitable for Heterogeneous Cell Populations

**Fig. 4:**
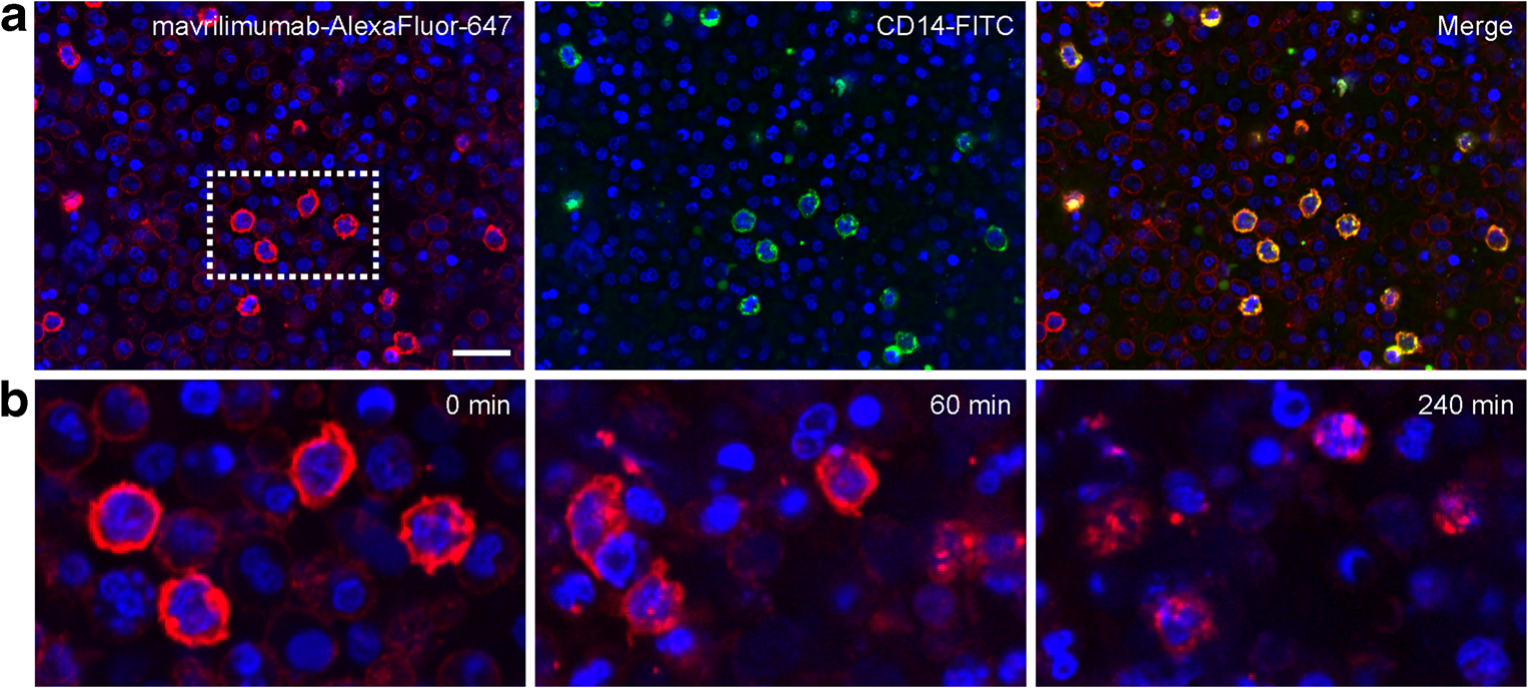
Mavrilimumab-AlexaFluor-647 internalization in human blood. (**a**) Image overlays of cells prior to internalization: left – Hoechst (*blue*) and mavrilimumab-AlexaFluor-647 (*red*); middle – Hoechst and CD14-FITC (*green*) and right - Hoechst, mavrilimumab-AlexaFluor647 and CD14-FITC. Colocalized CD14-FITC and mavrilimumab-AlexaFluor-647 are shown in yellow. (**b**) Mavrilimumab-AlexaFluor-647 internalization at time 0, 60 min and 240 min. A close-up view of the cells from the dotted frame shown in the image (a) is displayed. Scale bar, 25 µm.

We also examined internalization in whole blood samples, which could be useful when cell lines expressing the target are not available and when the target is expressed in peripheral blood. Figure 4 shows staining (a) and internalization (b) of mavrilimumab-AlexaFluor-647 in white blood cells from crude preparations of heparin anti-coagulated human blood. High intensity AlexaFluor-647 signals were associated with CD14^+^ cells (monocytes), which represented low-abundance cells in the total white blood cell population. This result was consistent with concurrent analysis of the sample by flow cytometry (data not shown). Mavrilimumab-AlexaFluor-647 translocation from the cell surface to cytoplasm was monitored continuously over time at 37°C and antibody internalization was clearly demonstrated (Fig. 4b). The results indicate that antibody internalization kinetics can be determined by this method using heterogeneous cell populations.

#### The Method is Suitable for Cells with Low Receptor Expression Levels

To test the ability of the method to measure k_int_ in cells expressing low levels of target receptors, we examined internalization of a mouse monoclonal antibody MMHAR2 directed against human interferon alpha/beta receptor 2 (IFNAR2) in THP-1 cells, known to express approximately 5,000 interferon receptors per cell [44]. Internalization T ½ was determined to be approximately 110 ± 30 min (n = 2). Results demonstrated that this method can measure internalization kinetics in cells with receptor expression levels as low as 5,000 receptors per cell.

#### The Method is Suitable for Monitoring Ligand Internalization

We examined if the developed method was amenable to assess k_int_ of ligands bound to cell surface receptors. GM-CSF is the agonistic ligand for GM-CSF receptor alpha. Similar to mavrilimumab, GM-CSF was chemically conjugated with AlexaFluor-647 and tested for internalization in TF-1 cells. GM-CSF-AlexaFluor-647 demonstrated faster internalization kinetics than the antibody with T ½ calculated to be 11 ± 4 min (n = 2). The internalization rate constant for GM-CSF-AlexaFluor-647 was also evaluated in a GM-CSF receptor over-expressing cell line, FD-hGMR. The T ½ of GM-CSF-AlexaFluor-647 internalization was 8 ± 2 min (n = 3), which was comparable to that estimated using TF-1 cells.

### Translational PK-PD Simulations

**Fig. 5:**
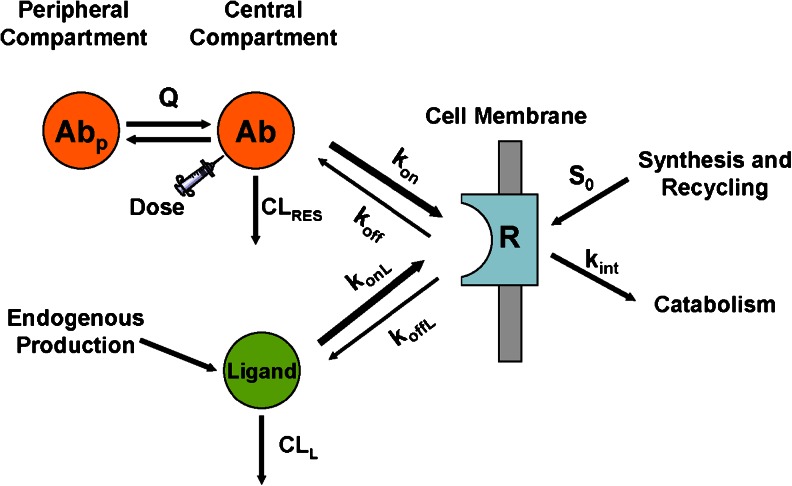
Mechanistic model structure for PK, target receptor occupancy and ligand displacement following antibody administration. The antibody (*Ab*) and an endogenous ligand compete for the binding to the target receptor (*R*). Upon binding to the receptor, the antibody-receptor and ligand-receptor complex are subsequently internalized and degraded inside the cells as characterized by rate constants k_int, AbR_ and k_int, LR_, respectively. Dose represents the intravenous administration of the antibody into the central compartment. Q is the inter-compartmental flow. CL_RES_ and CL_L_ are the systemic clearance of the antibody by the reticuloendothelial systems and that of the endogenous ligand, respectively. S_L_ and S_0_ are the zero-order endogenous production rate of the ligand and the target receptor, respectively. k_on_ and k_onL_ are the association rate constants for antibody and ligand, respectively. k_off_ and k_offL_ are the dissociation rate constants for antibody and ligand, respectively.

Biopharmaceuticals targeting cell surface receptors are frequently subject to target-mediated clearance, resulting in nonlinear pharmacokinetic profiles. To investigate the impact and clinical relevance of the internalization kinetics, a mechanistic model was constructed and used to simulate the pharmacokinetics of an antibody against a cell membrane receptor, the receptor occupancy, and displaced ligand levels in humans. In this model, the antibody and an endogenous ligand compete for the binding to the target receptor. Upon binding to the receptor, the antibody-receptor or ligand-receptor complex is subsequently internalized and degraded inside the cells (Fig. 5). The model allows for different k_int_ for the antagonistic antibody and agonistic ligand, and consequent alteration in the total amount of receptors on cell membrane. For example, when the internalization rate of antibody-bound receptors is much slower than that of ligand-bound receptors, the presence of antibody will effectively slow down the apparent receptor internalization process, resulting in an elevated level of receptors on the cell membrane surface. The model can also account for competition for receptor occupancy by elevated levels of the displaced, agonistic ligand.

**Fig. 6:**
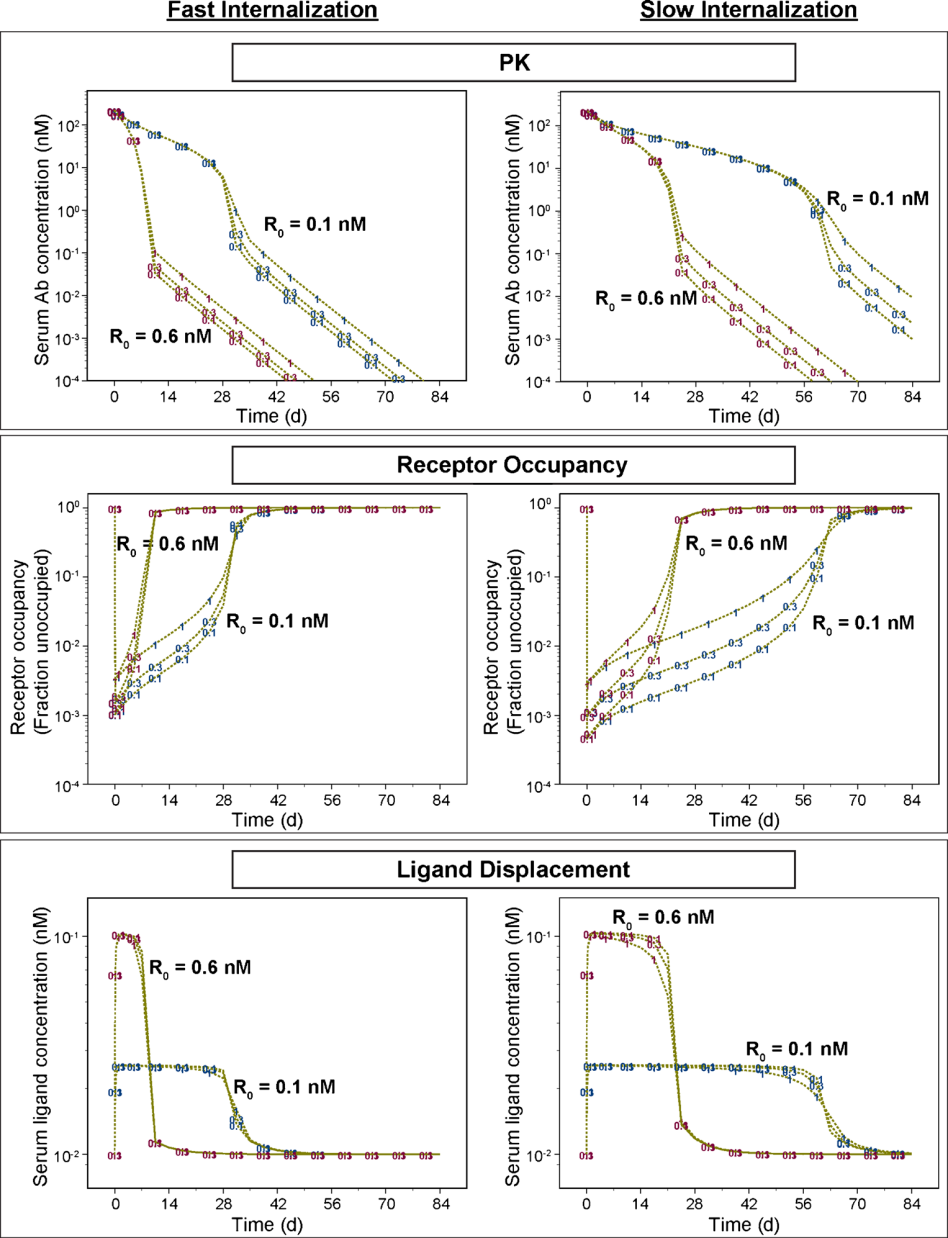
Simulated profiles of antibody PK, target receptor occupancy and displaced ligand in humans following a single 100 mg IV administration of an antibody directed against a cell surface target receptor. Fast- and slow-internalization rates correspond to receptor internalization T ½ of 30 min and 2 h, respectively. The presumed affinity of the endogenous ligand to receptor is 0.01 nM. Symbols represent the antibody affinity (Kd = 0.1, 0.3 or 1 nM). R_0_ is the expression level of the receptor at baseline. As shown in these graphs, target receptor expression level and internalization rate are two primary determinants of *in vivo* antibody PK, target receptor occupancy, and displaced ligand profiles.

Translational simulations demonstrated that the internalization rate, target receptor expression levels, and, to a lesser extent, antibody binding affinity, accounted for the nonlinear profiles of antibody PK, target receptor blockade and displacement of the endogenous ligand (Fig. 6). Serum levels of both antibody and the ligand decreased rapidly when the unoccupied (free) receptor level recovered to approximately 10% of the baseline. More rapid internalization or a higher expression of the target receptor is associated with more pronounced receptor-mediated clearance. An antibody with a higher affinity (lower K_d_) maintains >99% target receptor blockade for a longer period of time and enhances receptor-mediated clearance slightly. However, this effect diminishes when internalization is fast. These simulations demonstrate that a quantitative assessment of internalization rate can be important for the evaluation of target receptor “druggability”, protein engineering goals for affinity and pharmacokinetic properties, and the projected clinical dosing regimen.

## Discussion

We have developed a robust method for quantitative determination of k_int_ for biopharmaceuticals targeting cell membrane receptors by using high speed live-cell confocal imaging technology complemented with an image analysis algorithm. Receptor-mediated internalization is commonly observed when targeting cell surface receptors and the k_int_ is an important parameter that can be incorporated into mechanistic models describing the relationships between pharmacokinetics, receptor occupancy and displaced endogenous receptor ligand upon drug administration.

The use of most physiologically relevant conditions is important for assessment of internalization. Cells with endogenously expressed receptors are preferred target cells for evaluating internalization kinetics. However, endogenous receptor expression in many primary cells and cell lines may be as low as 2,000 – 5,000 receptors per cell, presenting technical challenges to internalization measurements. Our methodology using laser-driven fluorophore illumination and high resolution confocal imaging allowed measurement of internalization kinetic of interferon receptor in THP-1 cells expressing approximate 5,000 receptors per cell, demonstrating the suitability of the method for cells expressing endogenous receptors.

Although cells expressing endogenous receptors are favored due to their physiological relevance, cell lines transfected with recombinant receptors at high level often alleviate the problem of assay sensitivity. However, an engineered cell line might not be representative of the internalization kinetics of cells expressing endogenous receptors. Comparable rate constants of GM-CSF internalization in TF-1 cells expressing endogenous GM-CSF receptors with that in FD-hGMR cells over-expressing receptor provided confidence in using a transfected cell line for internalization measurements of GM-CSF receptor. However, appropriate selection of a cell line for transfection with recombinant receptors is critical, as not every cell line should be expected to possess appropriate endocytic machinery. In addition, upon internalization, overexpressed receptors may saturate endocytic capacity [45], leading to inaccurate estimation of internalization rate constants.

Multi-color imaging analysis allowed monitoring mavrilimumab-AlexaFluor-647 internalization in CD14^+^ monocytes using crude white blood cell preparations without enrichment, demonstrating applicability of the developed method to primary cells. Although primary cells are the most relevant physiological system, they present many challenges. Primary cells might express low levels of target receptors and also represent low-abundance cells in a mixed cell population. Multiple image fields should be acquired to obtain sufficient cell numbers for low-abundance cell populations. High speed image acquisition and multi-color imaging capability are critical for successful measurements of antibody internalization in low-abundance cells of a mixed cell population.

Because therapeutic targets are diversely expressed in various types of cells, the development of a method amenable to suspension and adherent cultures is imperative. Our versatile approach allowed k_int_ determination in both adherent and suspension cells. Adherent cells are more difficult in image analysis due to multi-layer topology and heterogeneity of cell morphology. Cell layers could change and detach during extended monitoring of internalization leading to out-of-focus, low quality images. In such cases, an alternative method is to detach adherent cells and to maintain them in suspension culture during internalization assessment. A comparable T ½ of MEDI-575 internalization in H1703 cells determined using adherent and suspension protocols, demonstrated that the method was amenable for both cell culture conditions. Therefore, if the target cells are an adherent cell line, an adherent protocol should be performed for greater physiological relevance. Alternatively, the suspension protocol could be also considered for practical and convenient purpose.

The confocal imaging methodology described offers a number of advantages over existing internalization methods. This approach measures internalization directly rather than the outcome of internalization. Direct measurement is generally preferred over indirect methods, which present various limitations. For example, an acid dissociation protocol relies on the efficiency of acid dissociation for accurate quantification of internalization. Many factors could impact the efficiency of acid dissociation and in turn affect accuracy of quantification. For example, some antibodies failed to completely dissociate from the surface receptors even in the presence of acid [37]. High affinity interactions, often observed for therapeutic antibodies, may lead to inefficient acid dissociation [46] and thus, to compromised internalization results. Acid dissociation protocols can be used for end point analysis but are not amenable for kinetic measurements of live cells. In addition, acid treatments could affect the cell viability and thus the accuracy of the analysis. A recently established antibody-toxin approach was based on inhibition of cell proliferation upon antibody internalization and toxin-mediated cell-killing. This approach yielded high assay sensitivities attributing to a high potency of toxins [35]. This assay, however, is more applicable for antibody screening rather than internalization rate constant measurement, because toxin functional activity is dependent on the cell duplication time and may not be in a linear relationship with efficiency of internalization.

Our method is capable of continuous monitoring antibody internalization in live cells and is a direct measurement of internalization. It monitors the same group of cells over the time course of internalization, significantly reducing variability. Moreover, high speed image acquisition allows recording of fast internalization kinetics and collecting kinetic images from multiple imaging fields and from replicate wells to obtain sufficient cells for accurate internalization analysis. There are a number of advanced live-cell fluorescence microscopes, which can also continuously monitor live-cell internalization. However, without fast image processing, these instruments are not capable of capturing rapid internalization kinetics in live cells. In order to acquire cell images at multiple time points within very short time intervals, cell fixation is required, resulting in only end-point measurements. Combination of fluorescence microscopy with flow cytometry in such systems as ImageStream [47] attained the capabilities of faster sample processing and image acquisition for larger cell numbers. However, even these advanced systems were still limited to end point measurements and were restricted to suspension cells.

Internalization of a biopharmaceutical upon binding to a cell surface target is the underlying cause of target-mediated drug clearance. Clinical implications include nonlinear PK when target is not fully occupied, a high dose or frequent dose administration to maintain target engagement, impractical subcutaneous administration due to high dose requirements, and an economically unviable cost of goods. The internalization kinetics can also affect the mechanism of action, such as the efficiency ADC payload delivery to tumors, tumor penetration, ability to elicit ADCC and CDC, and receptor upregulation or downregulation. Given the lengthy and costly drug development process, evaluation and selection of the optimal therapeutic target and drug candidate at the preclinical stage is imperative to reduce the development risk and increase the probability of success. For cell surface targets, the internalization rate is critical for evaluating the drug target and designing the optimum drug characteristics.

As demonstrated by translational simulations using a mechanistic model, a cell surface target at low occupancy can effectively eliminate the drug and endogenous ligand *via* target-mediated clearance. Time to this threshold greatly depends on the target expression level and the internalization rate. If >99% of target blockade by a biopharmaceutical is required for maximal target modulation, a higher binding affinity will lead to a more substantial and prolonged pharmacodynamic effect. However, the benefit of a higher affinity diminishes when the target-mediated clearance is very rapid (rapid internalization or high expression of the target). While the systemic expression level of a target *in vivo* might not be quantifiable until data are available from an animal model, the internalization rate can be readily studied *in vitro* using a quantitative imaging method. This robust and sensitive method has been successfully utilized in the evaluations of a therapeutic target and antibody affinity goal [26], characterizing the nonlinear PK profiles of an antibody against GM-CSF receptor alpha in RA patients [48], and clinical bridging of an anti-IFNAR antibody across two disease populations [49]. The quantitative measurement of internalization rate and advanced mechanistic PK-PD modeling are the foundation of all these model-based drug discovery and development practices.

## Conclusion

A robust and versatile confocal imaging method has been developed for measurement of internalization rate constants for therapeutic antibodies and other biopharmaceuticals, such as receptor ligands. This method offers advantages over other published approaches and is amenable to a broad range of cell types. Internalization assessment provides useful information for selection of drug target and for defining some of the optimal characteristics of biopharmaceuticals, including affinity and pharmacokinetic properties. The internalization rate constant is an important parameter for mechanistic PK-PD modeling, which has become an integral part of drug development. The combination of internalization information and mechanistic modeling provides valuable information for rational drug design and preclinical and clinical drug development.

## Electronic supplementary material

Below is the link to the electronic supplementary material.

Supplementary Fig. 1 (DOCX 60 kb)

Supplementary Table I (DOCX 13 kb)

## Abbreviations

ADC: Antibody-drug conjugate
ADCC: Antibody-dependent cell-mediated cytotoxicity
CDC: Complement-dependent cytotoxicity
CFSE: Carboxyfluorescein succinimidyl ester
CL_L_: Systemic clearance of the antibody by endogenous ligand
CL_RES_: Systemic clearance of the antibody by reticuloendothelial systems
IV: Intravenous
k_int_: Internalization rate constant
k_int,AbR_: Internalization rate constants for antibody-receptor complex AbR
k_int,LR_: Internalization rate constants for ligand-receptor complex LR
k_int,R_: Internalization rate constants for unbound receptor R
k_off_: Dissociation rate constants for antibody
k_offL_: Dissociation rate constants for ligand
k_on_: Association rate constants for antibody
k_onL_: Association rate constants for ligand
mAb: Monoclonal antibody
NaN_3_: Sodium azide
PK-PD: Pharmacokinetic-pharmacodynamic
Q: Intercompartmental flow
R: Receptor
T ½: Half-life
V_c_: Central distribution volumes
V_p_: Peripheral distribution volumes
