# Supplementary material for: Quantitative Measurement of the Target-Mediated Internalization Kinetics of Biopharmaceuticals
Source: Pharm Res. 2014 Sep 11;32(1):286–99. doi: 10.1007/s11095-014-1462-8 (PMC4284384; doi:10.1007/s11095-014-1462-8)
Supplement: Supplementary file 1 — (DOCX 60 kb) [file 11095_2014_1462_MOESM1_ESM.docx]

**Supplementary information**

**
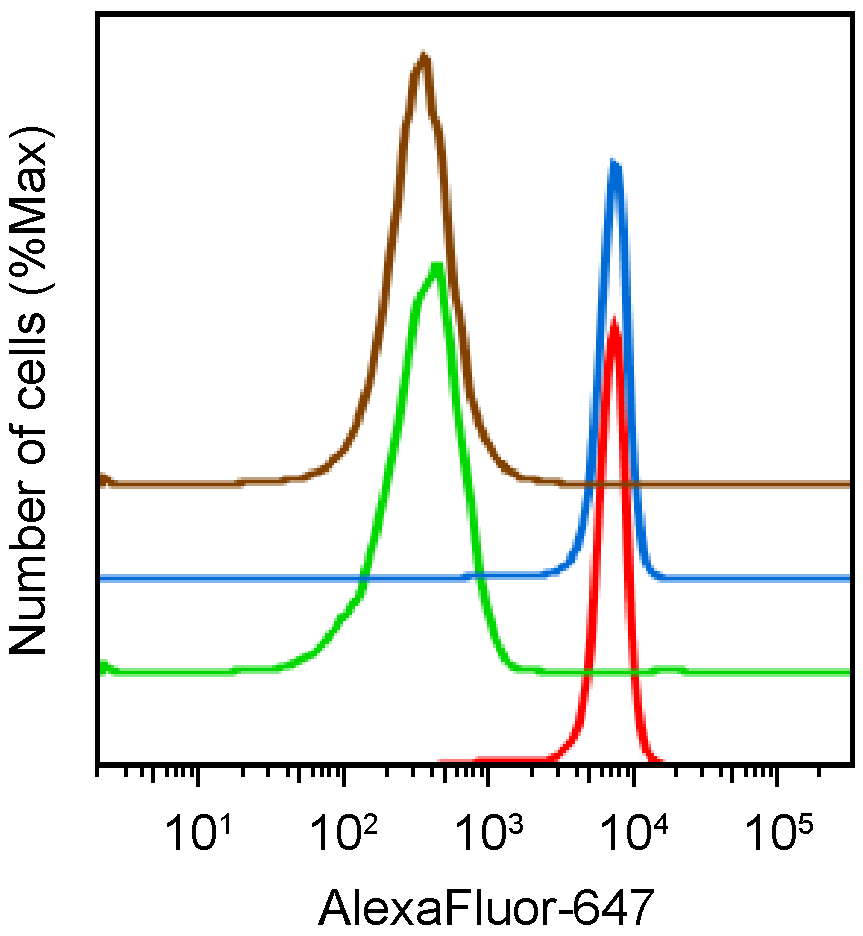
**

**Supplementary Fig. 1** Specificity of Mavrilimumab-AlexaFluor-647 binding to CD14^+^ human monocytes. Human heparinized blood was stained with Mavrilimumab-AlexaFluor-647 and CD14-FITC and analyzed using flow cytometry. Displayed are AlexaFluor-647 histograms in the CD14^+^ monocyte gates of the samples stained with Mavrilimumab-AlexaFluor-647 (red) or IgG-AlexaFluor-647 (green). To demonstrate specific binding, cells were pre-treated with unlabeled Mavrilimumab (brown) or IgG (blue) prior to addition of Mavrilimumab-AlexaFluor-647.
