# Supplementary material for: Quantitative Measurement of the Target-Mediated Internalization Kinetics of Biopharmaceuticals
Source: Pharm Res. 2014 Sep 11;32(1):286–99. doi: 10.1007/s11095-014-1462-8 (PMC4284384; doi:10.1007/s11095-014-1462-8)
Supplement: Supplementary file 2 — (DOCX 13 kb) [file 11095_2014_1462_MOESM2_ESM.docx]

**Supplementary Table I**

**Comparison of fluorescence signals generated by AlexaFluor-conjugates of Mavrilimumab upon binding to GM-CSF receptor alpha expressing cells using flow cytometry**

|  | **TF-1** | **FD-hGMR** |
| --- | --- | --- |
| **Mavrilimumab-AlexaFluor-488** | 2.6±0.4 | 4.4±1.3 |
| **Mavrilimumab-AlexaFluor-647** | 12.6±0.4 | 42.2±8.0 |

Antibody binding is expressed in folds of Mavrilimumab-AlexaFluor geometric mean values over the respective isotype controls (IgG-AlexaFluor-488 or IgG-AlexaFluor-647). Results were presented as mean ± standard deviation from 2 independent experiments.
